# Blood-tumor barrier in focus - investigation of glioblastoma-induced effects on the blood-brain barrier

**DOI:** 10.1007/s11060-024-04760-w

**Published:** 2024-08-28

**Authors:** Sanjana Mathew-Schmitt, Matthias Peindl, Philipp Neundorf, Gudrun Dandekar, Marco Metzger, Vera Nickl, Antje Appelt-Menzel

**Affiliations:** 1 Chair Tissue Engineering and Regenerative Medicine, University Hospital Würzburg, Röntgenring 11, 97070 Würzburg, Germany; 2 Translational Centre Regenerative Therapies TLC-RT, Fraunhofer Institute for Silicate Research ISC, Röntgenring 11, 97070 Würzburg, Germany; 3 Section Experimental Neurosurgery, Department of Neurosurgery, University Hospital Würzburg, Josef-Schneider-Straße 11, 97080 Würzburg, Germany

**Keywords:** Glioblastoma (GBM), Blood-tumor barrier (BTB), Blood-brain barrier (BBB), Human induced pluripotent stem cells (hiPSCs), Spheroids, Preclinical testing, In-vitro models

## Abstract

**Purpose:**

Glioblastoma (GBM) is the most prevalent, malignant, primary brain tumor in adults, characterized by limited treatment options, frequent relapse, and short survival after diagnosis. Until now, none of the existing therapy and treatment approaches have proven to be an effective cure. The availability of predictive human blood-tumor barrier (BTB) test systems that can mimic in-vivo pathophysiology of GBM would be of great interest in preclinical research. Here, we present the establishment of a new BTB in-vitro test system combining GBM spheroids and BBB models derived from human induced pluripotent stem cells (hiPSCs).

**Methods:**

We co-cultured hiPSC-derived brain capillary endothelial-like cells (iBCECs) with GBM spheroids derived from U87-MG and U373-MG cell lines in a cell culture insert-based format. Spheroids were monitored over 168 hours (h) of culture, characterized for GBM-specific marker expression and treated with standard chemotherapeutics to distinguish inhibitory effects between 2D mono-culture and 3D spheroids. GBM-induced changes on iBCECs barrier integrity were verified via measurement of transendothelial electrical resistance (TEER), immunocytochemical staining of tight junction (TJ) proteins claudin-5 and occludin as well as the glucose transporter-1 (Glut-1). GBM-induced secretion of vascular endothelial growth factor (VEGF) was additionally quantified.

**Results:**

Our hypothesis was validated by reduced expression of TJ proteins, occludin and claudin-5 together with significant barrier breakdown in iBCECs after only 24 h of co-culture, demonstrated by reduction in TEER from 1313 ± 265 Ω*cm^2^ to 712 ± 299 Ω*cm^2^ (iBCECs + U87-MG) and 762 ± 316 Ω*cm^2^ (iBCECs + U373-MG). Furthermore, 3D spheroids show more resistance to standard GBM chemotherapeutics in-vitro compared to 2D cultures.

**Conclusions:**

We demonstrate the establishment of a simplified, robust in-vitro BTB test system, with potential application in preclinical therapeutic screening and in studying GBM-induced pathological changes at the BBB.

## Introduction

Glioblastoma (GBM) is the most prevalent primary brain tumor in adults, with an incidence rate of 3.19–4.17 cases per 100,000 persons, with a 5-year survival rate of only 6.9% [1]. Standard therapeutic approaches entail aggressive surgical interventions aimed at the feasibility of preserving critical brain functions. Surgery is coupled with a regimen of radiotherapy, accompanied by adjuvant chemotherapy with Temozolomide [2]. As one option in second line therapy, recent treatment paradigms have been adapted to incorporate Lomustine for patients below the age of 70 and harboring a methylated MGMT (O6-methylguanine-methyltransferase)-promoter in their tumors [3]. Despite these refinements, disease survival landscape has remained strikingly unaltered since 2005, highlighting the dire need for novel therapeutic strategies [4, 5].

The cerebral endothelium, also known as the blood-brain barrier (BBB) comprises of specialized brain capillary endothelial cells (BCECs) that are connected to each other via dense tight junctions (TJs). The main functions of the BBB are divided into three subgroups, the physical-, metabolic- and transport-barrier. It chiefly serves to maintain central nervous system (CNS) homeostasis and protects the brain against neurotoxic substances and pathogens [6, 7]. However, for the effectiveness of drugs pertaining to neurological diseases, the restrictive nature of the BBB poses a problem. Numerous drugs cannot overcome the BBB in sufficient concentrations to reach the target location or they are metabolized prior to brain entry, thus becoming ineffective [8].

As GBM is characterized to be the most vascularized type of brain tumors, the BBB does not only play a role in tumor formation, growth and nutrient supply but also in effective therapy. The tumor brain vasculature is highly heterogeneous. Intact as well as leaky regions coexist due to constant remodeling of the extracellular matrix by tumor cells [9]. Although there is evidence of regional BBB disruption in GBM, most infiltration of therapeutics into the brain is protected by an intact BBB. Regional BBB disruption leads to formation of the BTB. While the BTB is more permeable than the healthy BBB, its heterogeneous permeability for therapeutics contributes to sub-optimal perfusion and accumulation of drugs, impairing their effects at the tumor site. Therefore, the BBB is a severe rate-limiting factor for clinical effectiveness [10].

Upon the onset of GBM, there is upregulated secretion of pro-inflammatory chemokines and growth factors. The consequence of this secretion from the tumor microenvironment primes and controls BCECs metabolism and function. In-vivo, this is characterized by reduced expression of TJ proteins, increased chemokine dependent cellular migration, breakdown of the basement membrane and disorganized astrocytic endfeet coupled with astrogliosis. This series of events further leads to increased BBB permeability. From a morphological aspect, BCECs lose their physiological phenotype towards the presentation of large nuclei and abundant cytoplasm [11].

CNS drugs have much lower approval rates at the Food and Drug Administration (FDA) than non-CNS drugs, with 45% CNS drugs failing to show improved efficacy over placebos. One reason behind this is the extensive failure of compounds in Phase III human clinical trials despite performing well in preclinical studies [12]. Clinical studies show that post 12 months of treatment with combined radiotherapy and Temozolomide tumor progression was observed in 43 out of 62 patients [13]. Several factors such as poor pharmacokinetic drug properties, missing complex tumor microenvironments, preclinical test model variability and heterogeneity are responsible for this failure, highlighting the importance of investigating BBB permeability/breakdown in human-specific models to improve the predictive power of preclinical testing.

Three major types of existing preclinical GBM in-vivo models are (1) chemically induced models, (2) xenograft models, and (3) genetically engineered mouse models [12]. Species-based differences as well as the substitution of BBB-specific cell sources with divergent functional characteristics such as epithelial cell lines or endothelial cells of non-cerebral origin could be a major factor adding to high rates of clinical drug failure. Advancements in therapy options are therefore highly dependent on the availability of validated, reliable and robust model systems [14].

Due to high demands in basic and preclinical research, a range of in-vitro BBB models have been developed, out of which the human induced pluripotent stem cell (hiPSC)-derived brain capillary endothelial-like cell (iBCEC) models have shown clinical relevance in translational studies, investigating bacterial [15] or viral infections [16] and BBB permeability to several therapeutics [17, 18]. These in-vitro models are effective in bridging the gap between animal-based and existing cell line-based studies. Main characteristics of this system are in-vivo-like trans electrical endothelial resistance (TEER) values up to 2,500 Ω*cm^2^, the presence of a complex TJ networks and a strong upregulation of typical BBB genes [19, 20]. The complexity of this in-vitro BBB model can be increased by including other neurovascular unit cell (NVU) types.

To study and validate therapeutic GBM approaches, human-specific preclinical test systems are required that can reflect complex in-vivo physiology. For this, in this study we combined previously established iBCECs together with three dimensional (3D) GBM spheroids to generate a simplified in-vitro BTB test system which allows monitoring of changes occurring to the BBB upon onset of GBM. We were able to successfully identify that upon co-culture with 3D GBM spheroids, iBCECs show significant barrier breakdown, similar to in-vivo observations. Furthermore, 3D GBM spheroids presented higher resistance towards treatment with standard GBM chemotherapeutics, which reflects similarity to the in-vivo situation.

## Methods

### Standard culture of glioblastoma cell lines

GBM cell lines U373-MG and U87-MG (purchased from CLS, Eppelheim, GER) were cultured in DMEM, high glucose, Gluta-MAX™ (Gibco™, 10569010 USA) supplemented with 10% fetal calf serum (PAN Biotech, P30-3306, GER), 1% Penicillin/Streptomycin (Gibco™, 15140122, GER), 1% L-Glutamine (Gibco Life Technologies, 2503008, GER), 1% Sodium-Pyruvate (Thermo Scientific™, 11360039, GER), and 1% MEM Non-essential amino acids (Gibco™, 11130051, GER). This medium is further referred to as GBM medium. Both cell lines were cultured under standard conditions at 95% humidity, 5% CO_2_ and 37 °C. After reaching a confluency of 70–80% the cells were passaged at 1:20 or 1:50 ratios. Media was renewed every second day.

### Generation of GBM spheroids

GBM spheroids were generated by seeding the cell lines U373-MG or U87-MG in either 24-well AggreWell™ 400 plates (STEMCELL™ Technologies, 34450, CAN) for the co-culture models or in round-bottom 96-well Ultra Low Attachment (ULA) plates (Costar^®^ Ultra Low Cluster, 96 Well, Round Bottom, CLS3474-24EA, US) for drug testing and spheroid growth observation, respectively. AggreWell™ plates were coated with 500 µL Anti-Adherence rinsing solution (STEMCELL™ Technologies, 07010, CAN) per well and centrifuged for 5 minutes (min) at 13,000 x g. The wells were washed once with cell specific medium before adding the single cell suspension. In each well, 1.2 × 10^6^ cells of the corresponding cell line were seeded in a final volume of 1.5 mL medium. The plate was centrifuged for 3 min at 100 x g. A partial medium change (1 mL) was performed daily only within AggreWell™ plates. For ULA plates, 1,000 cells were seeded per well in a final volume of 100 µL and the plates were centrifuged for 3 min at 100 x g. No medium changes were performed on ULA plates.

### Size measurement of GBM spheroids

To evaluate the longevity of GBM spheroids, diameter was measured over the cultivation time of 168 h. Brightfield images of 10 representative spheroids were recorded using Evos XL microscope (Life Technologies Corporation, USA) every 24 h of culture until day seven. According to the given scale, the Ferret’s diameter of the spheroids was measured with ImageJ’s (Wayne Rasband, National Institute of Health, V1.52a, US) measuring tool.

### Evaluation of chemotherapeutic efficiency in 2D and 3D

The potency of the chemotherapeutics Temozolomide (Absource Diagnostics GmbH, SKU: S1237-0025, GER) Lomustine (Absource Diagnostics GmbH, SKU: S1840-0050, GER) and Carmustine (Absource Diagnostics GmbH, SKU: S3669-0025, GER) was determined in-vitro by comparing their IC50-values in 2D GBM mono-cultures and 3D GBM spheroids. For this, CellTiter-Glo^®^ Luminescent Cell Viability Assay (Promega, G7570, GER) or CellTiter-Glo^®^ Luminescent 3D Cell Viability Assay (Promega, G9682, GER) were applied on the corresponding cultures following the manufacturer’s instructions. In brief, for 2D testing, single cells (11,000/cm^2^ U87-MG or 22,000/cm^2^ U373-MG) were seeded in 96-well plates (Greiner Bio-One, 655983, GER) in a total volume of 100 µL. For 3D spheroids, 1,000 cells were seeded per ULA well (Corning^®^, 4515, GER). The 2D and 3D cultures were pre-cultured for 24 h under standard conditions before treatment with increasing concentrations of the corresponding chemotherapeutics for up to 72 h. Only for 2D cultures, treatment was renewed after 48 h of initial treatment due to strong proliferation of the cells. Luminescence was recorded by aid of a microplate reader (TECAN Infinite M200, SWI; integration time = 1000 ms). Respective IC50-values were calculated with Prism (GraphPad Software, Inc., Version 9, US) based on the non-linear regression of the luminescence.

### Culture of human induced pluripotent stem cells (hiPSCs)

HiPSC line IMR90-4 (purchased from WiCell) was cultured in mTeSR™-1 medium (STEMCELL™ Technologies, 85,851, CAN) under adherent cell culture conditions on 100 µg/mL Matrigel™ (Corning, 356231, US)-coated 6-well plates (Nunc™, 140675, GER). Prepared coating solution was then dispensed as 1 mL/well and incubated for 1 h at room temperature (RT). When hiPSCs reached a confluency of 50–80% the cells were passaged via incubation with Gentle Cell Dissociation Reagent (STEMCELL™ Technologies, 100–0485, CAN) for 5 min at 37 °C. Cells were detached using a cell scraper with 2-position blade (SARSTEDT, 83.395, GER) and homogenized gently via pipetting and reseeded at a ratio of 1:6 − 1:8. The first 24 h in culture post passaging, hiPSCs were cultivated in mTeSR™-1 containing 10 µM Rho-Kinase inhibitor (ROCK-inhibitor) (Sigma-Aldrich^®^, 688000, GER). Standard culture was continued by replacing media with 2 mL per well of mTeSR™-1.

### Differentiation of hiPSCs to iBCECs

HiPSCs were differentiated to iBCECs as previously reported [21]. Briefly, hiPSCs were detached with 1 mL/well Accutase (Sigma-Aldrich^®^, A6964, GER) and seeded at a density of 7.5 × 10^3^ cells/cm^2^ onto Matrigel^TM^-coated 6-well plates. Seeding was performed in 2 mL/well mTeSR™-1 including 10 µM ROCK-inhibitor for the first 24 h. From the following day, medium was changed daily and hiPSCs were proliferated in 2 mL/well mTeSR™-1 without ROCK-inhibitor. Once hiPSCs reached a confluence of 2.5–3.5 × 10^4^ cells/cm^2^, co-differentiation into iBCECs was initiated by change to unconditioned medium (UM; DMEM/F-12 without L-Glutamine (ThermoFisher Scientific, 11330-057, GER), 20% KnockOut™-Serum Replacement (ThermoFisher Scientific, 11330-057, GER), 1% MEM Non-Essential Amino Acids (100X; ThermoFisher Scientific, 10828-028, GER), 1 mM L-Glutamine (Sigma-Aldrich^®^, G7513-100ML, GER), 0.1 mM β-mercaptoethanol (Sigma-Aldrich^®^, M3148-25ML, GER)). Medium was changed daily for the following 6 days. On day 6, medium was changed to EC++ (Human Endothelial Serum Free Medium (HESFM; ThermoFisher Scientific, 11111-044, GER) supplemented with 0.5% B27 (ThermoFisher Scientific, 17504-044, GER), 20 ng/mL human basic fibroblast growth factor (PeproTech, 100-18B, GER) and 10 µM retinoic acid (Sigma-Aldrich^®^, R2625-500MG, GER)). On day 7, no medium change was performed. On day 8, cells were passaged in EC + + media. Cells were detached via 30 min treatment with Accutase and reseeded in a density of 1 × 10^6^ cells/cm^2^ onto Matrigel^TM^-coated cell culture inserts (CellQART^®^, 932 04 02, GER; 200 µg/mL Matrigel™ in 100 µL/cell culture). Cells were seeded onto the apical compartment, such that the apical compartment consisted of 200 µL medium per cell culture insert and basolateral compartment contained 850 µL medium. For the following days in culture, iBCECs were cultivated in EC + media (HESFM supplemented with 0.5% B27). Differentiated iBCECs were used for further analysis and co-culture from day 10 onwards.

### Establishment of BTB model

U87-MG and U373-MG-derived spheroids were prepared three days prior to day 10 of iBCEC differentiation. On day 10 of iBCEC differentiation, cell culture inserts containing iBCECs were placed onto AggreWell™ plates containing pre-formed spheroids. Post this, media on the apical compartment of the insert was changed to 500 µL of GBM medium. No medium change was performed on the basolateral side. This set up was cultured under standard conditions at 95% humidity, 5% CO_2_ and 37 °C for 24 h before further analysis.

### Immunofluorescence staining

iBCECs on cell culture inserts were fixed with 4% paraformaldehyde (Applichem, A3813.1000, GER) for 10 min. For immunofluorescence staining of 3D spheroids, samples were fixed with 4% ROTI^®^Histofix (Carl Roth^®^, P087.2, GER) for 30 min at RT. The samples were washed three times for 5 min with PBS (Sigma-Aldrich^®^, D8537, GER) before adding 0.2% TritonX 100 (Carl Roth^®^, 3051.2, GER; prepared in PBS) for permeabilization. Permeabilization was performed for 20 min in iBCECs and 30 min in GBM spheroids, followed by 3x washing for each 5 min with 0.5% Tween-20 (VWR, 8.221.840.500, GER; in PBS). The samples were then blocked for 20 min with 5% donkey serum (iBCECs) or for 30 min with 10% (spheroids) donkey serum (Biozol, SBA-0030-01, GER) containing 0.02% saponin (Carl Roth^®^, 9622.1, GER) and 0.1% Triton-X 100. Antibodies were diluted in antibody dilution solution (DCS Innovative Diagnostik-Systeme, ALI20R500, GER) and added to the samples followed by incubation at 4 °C on an orbital shaker either overnight (iBCECs) or for 48 h (spheroids). Primary antibodies binding to the following proteins were applied: claudin-5 (Abcam, ab15106, UK), GFAP (Dako, Z0334, DNK), GLUT-1 (Abcam, ab40084, UK), nestin (Merck, MAB53226, USA), occludin (Thermo Fisher, 33-1500, USA) and SOX-2 (Abcam, ab137385, UK). Post incubation, samples were washed 3x for 5 min each with 0.5% Tween-20 in PBS. Secondary antibodies were diluted in a ratio of 1:400 using antibody dilution solution (DCS Innovative Diagnostik-Systeme, ALI20R500, GER) and incubated with the samples on an orbital shaker either for 2 h at RT (iBCECs) or for 24 h at 4 °C (spheroids). Post washing 3x for 5 min each, samples were mounted on a glass microscopy slide (iBCECs) or placed onto a 96 well glass bottom plate (Mobitec, 130-098-262, GER) and covered with Fluoromount-G™-Dapi (Biozol Diagnostica, SBA-0100-01, USA). 2D images were recorded with Microscope BZ-9000 BIOREVO System (Keyence, GER) and 3D images were recorded with CellVoyager™ CQ1 Benchtop High-Content Analysis System (Yokogawa, JPN).

### Transendothelial electrical resistance (TEER) measurements

Barrier integrity of iBCECs was measured with a Millicell^®^ ERS-2 System (Merck, USA). The instrument was first sterilized by placing electrodes in 70% ethanol (Sigma-Aldrich^®^, 51976, GER) for 15 min, followed by washing with sterile deionized water. 40 min prior to measurements, media was changed on cell culture inserts to allow for equilibration of the cells in the incubator. Background resistance was measured on Matrigel^TM^-coated blank cell culture inserts without cells. TEER of iBCECs were calculated by subtraction of blank values and multiplication with the culture area [cm^2^] of the cell culture inserts. For each cell culture insert, three measurements were performed at different positions of the insert.

### Analyses of VEGF secretion

Human vascular endothelial growth factor (VEGF) quantikine ELISA (R&D Systems, DVE00, USA) was performed according to the manufacturer’s instruction. In brief, the washing buffer and VEGF standards were brought to RT and prepared according to the instructions. The supernatants (basolateral site) of U87-MG or U373-MG mono- (post 96 h after seeding) and co-culture models (post 24 h of co-culture) were diluted as 1:10 or 1:25 in respective cell culture growth medium. A volume of 200 µL standards or sample analytes was added into the ELISA plate, followed by an incubation for 2 h at RT. Conjugates were added after washing thoroughly followed by incubation for 2 h at RT. Subsequently, the substrate was added, and the reaction was stopped after 20 min. The optical density at 450 nm with a correction wavelength of 540 nm were measured using a microplate reader (TECAN Infinite M200, SWI). VEGF concentrations were determined using OriginPro^®^ 2021 (OriginLab, version 9.8.5.212, USA).

### Statistical analysis

Statistical significance was determined by ANOVA. All results are indicated as mean ± standard deviation (SD). Significant results are indicated as follows: * *p*-value ≤ 0.05, ** *p*-value ≤ 0.01, *** *p*-value ≤ 0.001. Statistical tests were performed with Prism (GraphPad Software, Inc., Version 9, US).

## Results

### GBM spheroids are successfully formed in-vitro and show prolonged culture capacity

**Fig. 1:**
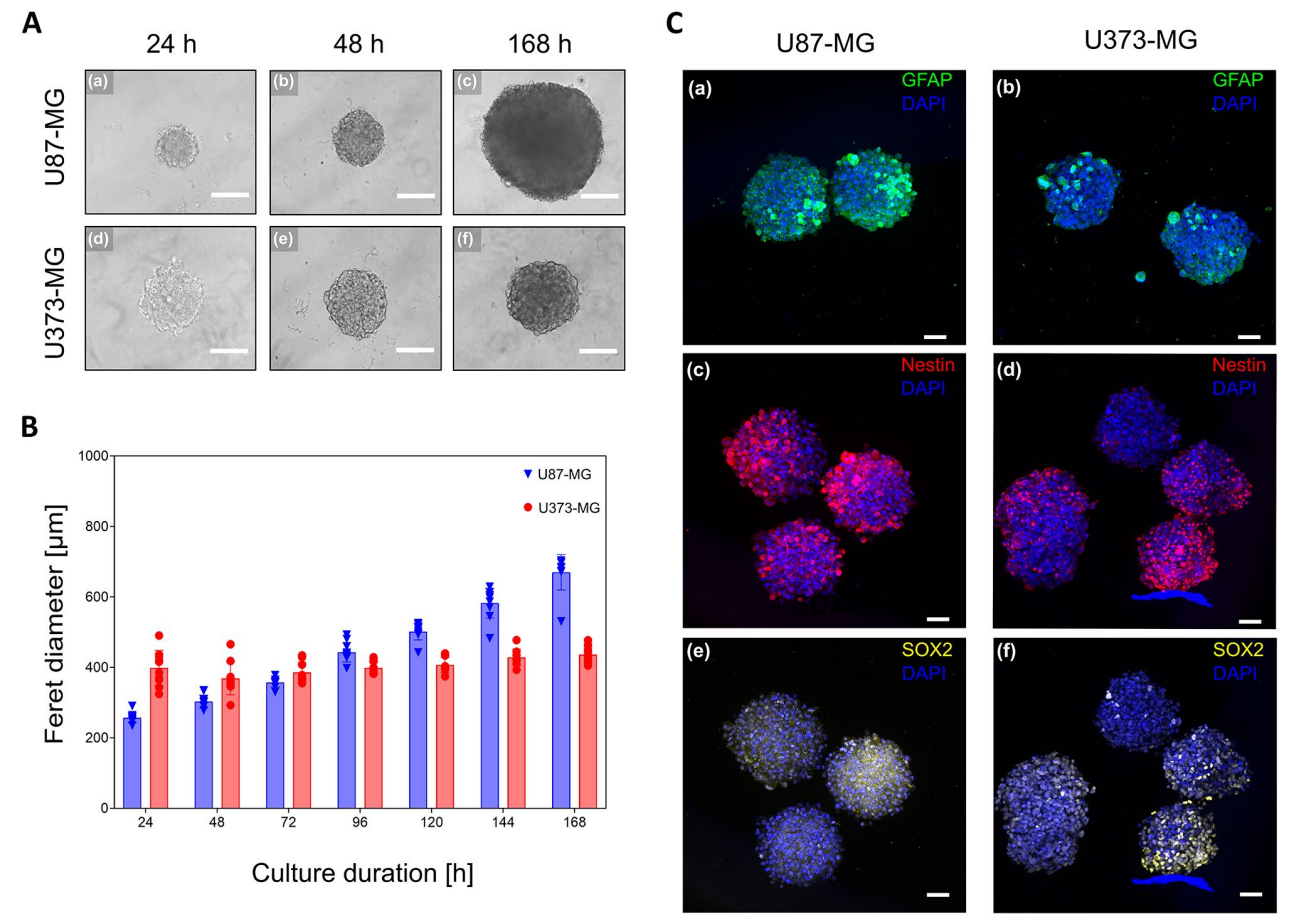
Characterization of glioblastoma (GBM) spheroids. (**A**) Brightfield images of U87-MG-derived spheroids and U373-MG-derived spheroids at 24 h (**a, d**), 48 h (**b, e**) and 168 h (**c, f**) post seeding. Scale bar = 200 μm, magnification = 10x. (**B**) Monitoring of measured Feret diameter (µm ± SD) of GBM spheroids for up to 168 h, *n* = 10 technical replicates per time point and GBM line. (**C**) Expression of glial fibrillary acidic protein (GFAP, **a, b**), nestin (**c, d**), and SRY (sex determining region Y)-box 2 (SOX2, **e, f**) was verified in GBM spheroids. Representative maximum projection images are presented, scale bar = 50 μm.

As we established a previously reported strategy to differentiate hiPSCs into iBCECs with high TEER [21], we aimed at combining iBCECs with GBM spheroids to reflect barrier breakdown as observed in-vivo. For better standardization, we established the BTB model with commonly used GBM cell lines. We tested the capacity of standard GBM cell lines U87-MG and U373-MG for spheroid formation and prolonged in-vitro culture. Both U87-MG and U373-MG lines formed spheroids within 24 h of seeding. While at 168 h, U87-MG-derived spheroids showed profound increase in size, U373-MG spheroids remained stable (Fig. 1, A). This was quantified via measurements of spheroid Feret diameters. U87-MG-derived spheroids demonstrated a mean diameter of 257 ± 13 μm at 24 h post seeding which increased to 685 ± 11 μm at time point 168 h. U373-MG-derived spheroids demonstrated a diameter of 394 ± 48 μm at 24 h post seeding, with a minor increase to 436 ± 22 μm at 168 h (Fig. 1, B). Additionally, we verified the expression of characteristic GBM markers in spheroids. In both spheroid types, nestin and glial fibrillary acidic protein (GFAP) were expressed (Fig. 1C, a-d). Importantly, fewer nestin and GFAP positive cells were observed in U373-MG-derived spheroids in comparison to U87-MG-derived spheroids. In order to determine the presence of glioma stem cells, we also stained for SRY (sex determining region Y)-box 2 (SOX2). It was observed that U373-derived spheroids showed characteristic nuclear expression of SOX2 in comparison to U-87-derived spheroids, presenting a more cytoplasm-like staining pattern (Fig. 1C, e-f).

### 3D spheroids show more resistance to standard GBM chemotherapeutics in-vitro

**Fig. 2:**
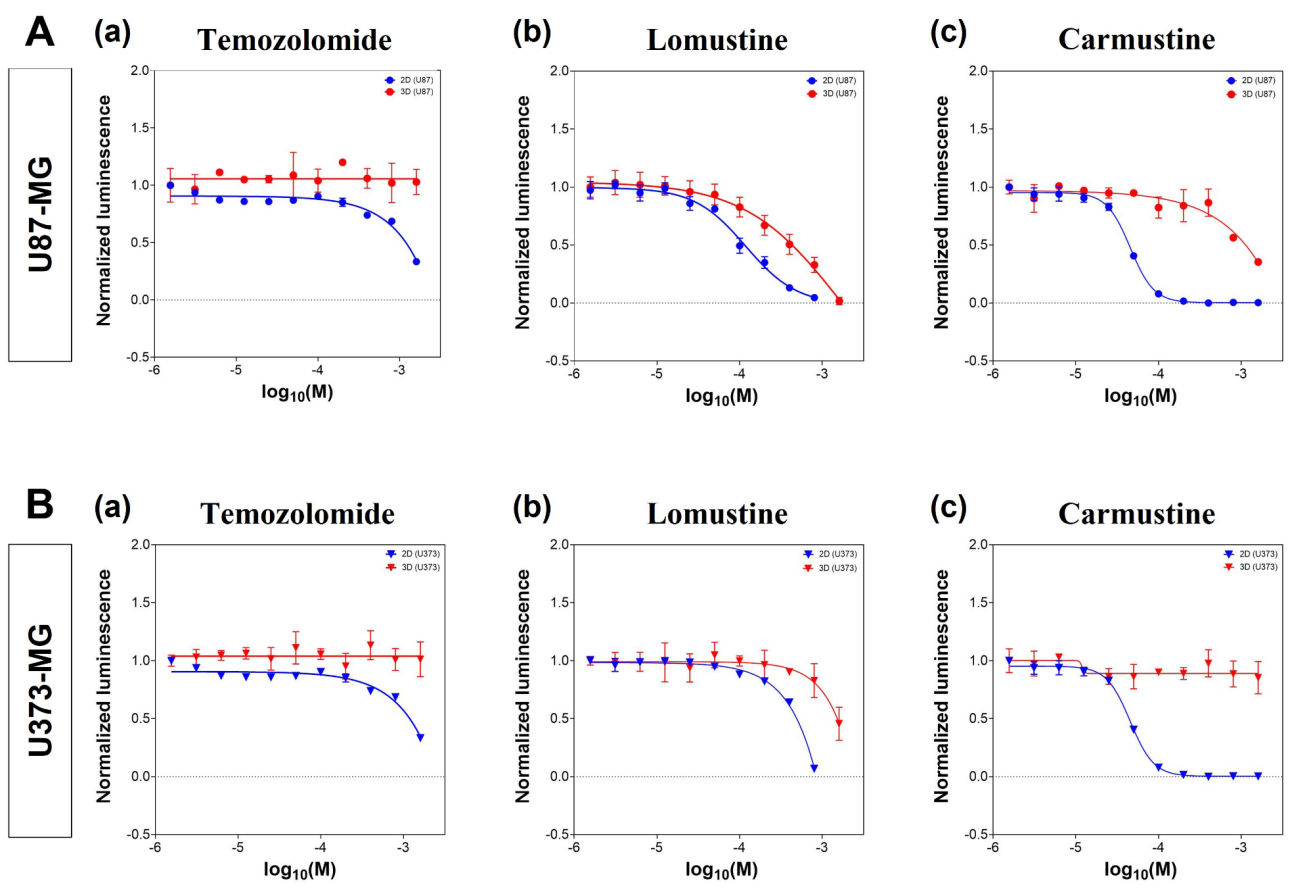
Drug response to standard chemotherapeutics Temozolomide, Lomustine, and Carmustine. (**A**, **a-c**) IC50-values of GBM therapeutics determined in 2D (blue line) as well as 3D GBM spheroid (red line) cultures of the GBM cell line U87-MG. (**B, a-c**) IC50-values of GBM therapeutics determined in 2D (blue line) as well as 3D GBM spheroid (red line) cultures of the GBM cell line U373-MG. *N* = 3 technical replicates in *n* = 2–3 biological replicates.

In order to validate the generated BTB model we aimed to determine the efficacy of standard GBM chemotherapeutics such as Temozolomide, Lomustine, and Carmustine to provide effective tumor killing. For this, IC50-values were determined in both 2D and 3D (Fig. 2). In contrast to 3D, U87-MG and U373-MG cell lines in 2D presented lower IC50-values for all tested compounds. Temozolomide reached IC50-values at 1171 µM in U87-MG-2D cultures while U87-MG-derived spheroids were resistant within applied concentration spectrum. Temozolomide IC50-values were additionally indeterminable for both 2D and 3D conditions of U373-MG. Lomustine achieved IC50-values of 108.2 µM in U87-MG-2D cultures and of 425.2 µM in U87-MG-derived spheroids. In U373-MG-2D cultures, Lomustine reached IC50 at 478.6 µM while in U373-MG-derived spheroids IC50 was achieved at 1484 µM. Carmustine attained IC50-values of 45.52 µM in U87-MG-2D cultures and of 1082 µM in U87-MG-derived spheroids. In U373-MG-2D cultures, Carmustine reached IC50 at 226.1 µM while in U373-MG-derived spheroids IC50 was indeterminable. In summary, the results show that the GBM line U87-MG is more sensitive to all three chemotherapeutic agents compared to the line U373-MG, in 2D as well as in 3D conditions. Furthermore, both GBM cell lines are more resistant to the tested agents in 3D conditions, rather than in 2D, demonstrating the value of 3D models in in-vitro testing.

### BBB disruption upon co-culture with GBM spheroids leads to formation of the BTB

**Fig. 3:**
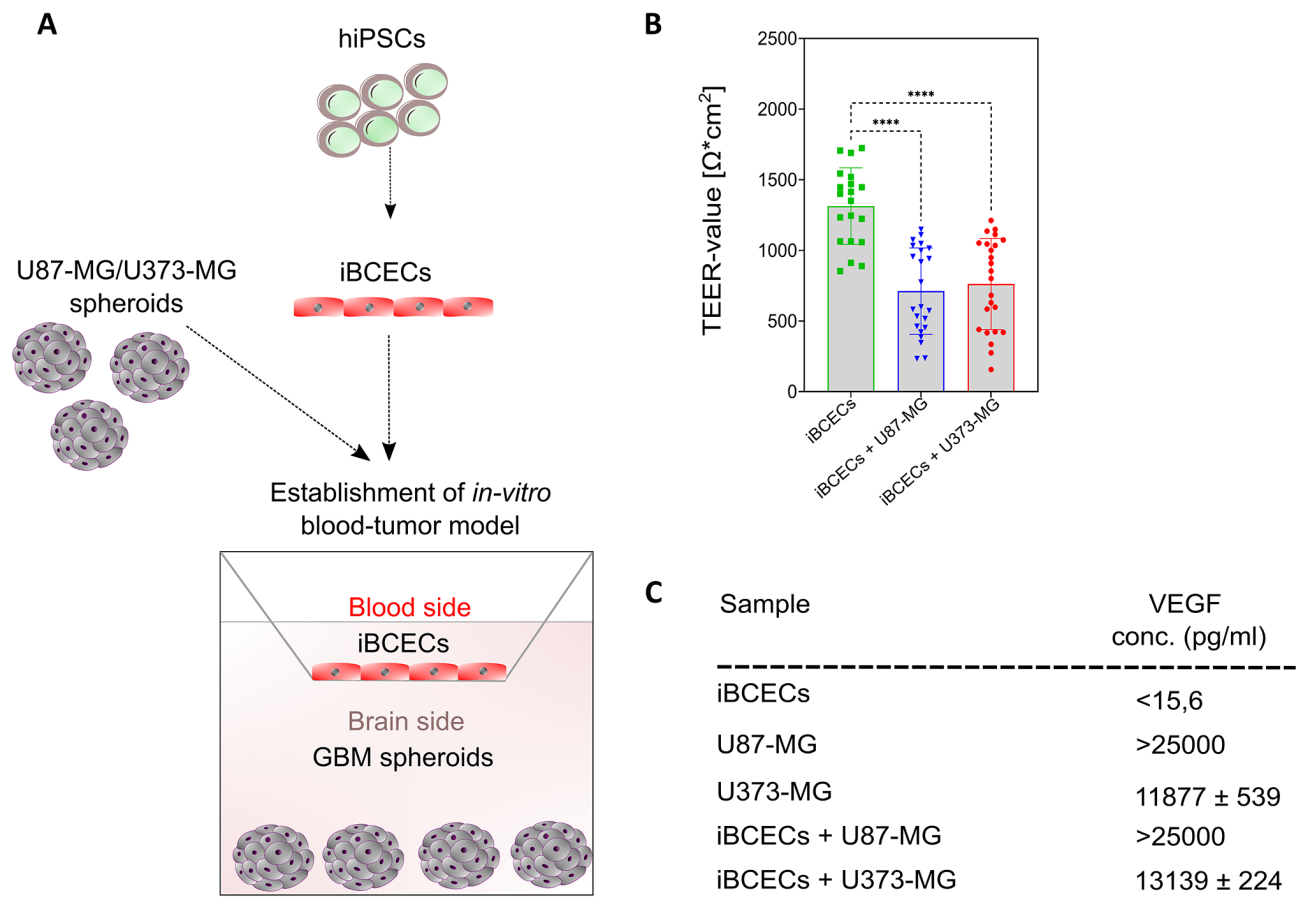
Blood-tumor barrier (BTB) establishment, outcomes and rational. (**A**) Schematic of BTB model establishment: Human induced pluripotent stem cells (hiPSCs) were differentiated to brain capillary endothelial-like cells (iBCECs). IBCECs, seeded on apical compartment of cell culture inserts, were combined with either U87-MG or U373-MG-derived GBM spheroids cultivated on the basolateral compartment. (**B**) Transendothelial electrical resistance (TEER [Ω*cm²]) of iBCECs represented as mean ± SD was significantly reduced due to GBM co-culture, *n* = 8 biological replicates in three biological independent assays. Analysis was performed via one-way ANOVA and **** = *p*-value ≤ 0.001. (**C**) Vascular endothelial growth factor (VEGF) concentrations from supernatants of mono-cultured U87-MG-derived spheroids, U373-MG-derived spheroids, iBCEC mono-cultures and BTB model co-cultures were analyzed using enzyme-linked immunosorbent assays (ELISA), *n* = 2 biological replicates in *n* = 2 independent biological assays.

**Fig. 4:**
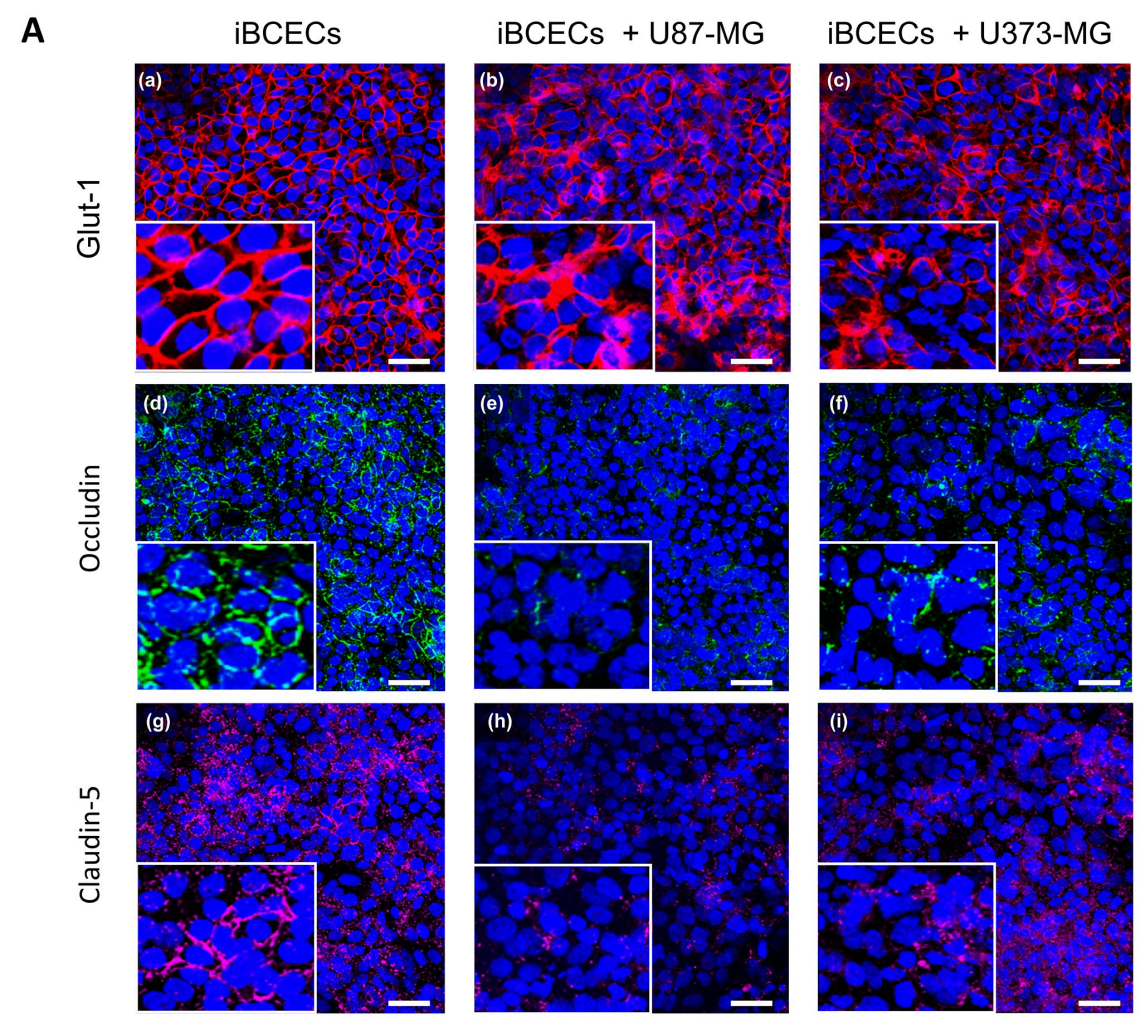
Expression patterns of characteristic blood-brain barrier (BBB) markers are influenced by glioblastoma co-culture. (**A**) Protein expression of BBB-relevant glucose transporter 1 (GLUT-1, **a-c**) as well as the tight junction proteins claudin-5 (**d-f**) and occludin (**g-i**) were compared in mono- and co-cultures. iBCECs stimulated by GBM co-culture were characterized by an altered expression pattern compared to corresponding mono-cultures. Scale bar = 50 μm, magnification 20 x, representative images of *n* = 2 biological replicates.

To generate BTB models, cell culture insert-based setups were used. iBCECs were cultured on top of the cell culture insert membrane so as to represent the ‘*blood side*’ and GBM spheroids were cultured in the bottom compartment in non-adhesive cell culture ware to represent the ‘*brain side*’ (Fig. 3, A). In order to identify, if barrier integrity of iBCECs is compromised due to co-culture with GBM spheroids, we investigated BBB disruption in iBCECs. We observed a significant decrease in functional barrier integrity, measured by TEER analyses starting from 1313 ± 265 Ω*cm^2^ in iBCECs mono-cultures which dropped down to 712 ± 299 Ω*cm^2^ (iBCEC + U87-MG spheroids) and 762 ± 316 Ω*cm^2^ (iBCEC + U373-MG spheroids) due to GBM spheroid co-culture (Fig. 3, B). Further, we identified that iBCECs stimulated by GBM co-culture were characterized by an altered and decreased expression pattern of TJ proteins. Occludin and claudin-5 expression was highly affected, indicated by a significant decrease and loss of characteristic cell border-specific localization of the proteins (Fig. 4, A, d-i). As tumors are primarily supplied with nutrients such as glucose for their proliferation, we also looked for variations in expression patterns of the BBB-relevant glucose transporter-1 (GLUT1). Here we observed loss of cell-border specific protein expressions post co-culture, which could represent abnormal functionality and iBCEC remodeling (Fig. 4, A, a-c). We additionally hypothesized that vascular endothelial growth factor (VEGF), secreted by the tumor cells could be one influencing factor resulting in barrier breakdown of iBCECs. By ELISA measurement of the supernatants in the different culture conditions, we were able to identify that under mono-culture conditions, U373-MG-derived spheroids secrete 11,877 pg/ml VEGF, while under co-culture with iBCECs they secrete 13,139 pg/ml VEGF. For U87-MG-derived mono-cultured spheroids and for co-culture with iBCECs, the amount of VEGF measured was well over 25,000 pg/ml (Fig. 3, C), indicating that excess VEGF secretion from GBM spheroids could be a factor responsible for compromised BBB in our test system. VEGF concentration in supernatants of iBCEC mono-cultures were much lower than least measurable values.

## Discussion

In this study, we combined a well-established BBB model using hiPSC-derived BCECs with highly resistant GBM spheroids to generate a simplified and reliable BTB in-vitro test system. The highlight of our study is the occurrence of barrier disruption of iBCECs induced by co-culture with tumour spheroids. This was indicated via decreased and dislocated TJ proteins expression and significant TEER value reduction compared to the corresponding controls.

Tissue-engineered models of GBM aim to offer tunable and high throughput pre-clinical screening tools compared to classical animal models. Primary and immortalized GBM cell cultures have been critical in generating widespread and accessible in-vitro models. However, a rare number of these systems are designed to include the BBB/BTB component, which plays a vital role in deciphering the efficacies of drug delivery in-vivo [22–24]. Compared to 2D glioma cultures, spheroids are more accurate in mimicking in-vivo physiology and are referred to as gold standards in this field [25]. One major reason for this is their characteristic to be maintained under long-term culture conditions, allowing prolonged high throughput monitoring of drug treatments. Importantly, not all tumor cell lines can form spheroids or if so, maintain their size or shape. In our studies, we observed differences between spheroids generated from two different GBM cell lines, U87-MG and U373-MG. Although both GBM lines formed homogenously spheroids, U87-MG-derived spheroids showed profound increase in size over the cultivation time of 168 h, while U373-MG spheroids did not. However, they still maintained their morphology with increased culture durations.

With the goal of investigating therapeutic efficacy, we compared drug responses between 2D and 3D culture conditions by dose-dependent treatment with standard chemotherapeutics Temozolomide, Lomustine, and Carmustine. Our analysis was based on the concentration of drug necessary to reduce tumor cell survival by 50% (determination of IC50-values). Here, we observed that standard GBM cell lines cultured as spheroids resulted in significant higher IC50-values. However, the difference in IC50-values observed in this study, is not clinically relevant due to high values measured in both culture systems. Furthermore, we specify the treatment regime between 2D and 3D cultures to prevent viabilities induced by starvation or a pH drift. The median IC50 of Temozolomide for U87-MG at 24 h, 48 h and 72 h of treatment is reported to be 123.9 µM, 223.1 µM and 230.0 µM, respectively [26]. Carmustine is reported to achieve IC50 values of 18.2 µM in 2D cultures of U87-MG cells [27].

Combining the BBB with GBM is chiefly reported on cell culture insert-based systems, with the usage of immortalized human brain capillary endothelial cells, such as hCMEC/D3 cell line, and 2D cultured GBM cell types [28]. iBCECs are generally used in determining drug permeability or brain pathogen interactions. Recently, they have found a new application in studying the role of the BBB in immune surveillance and inflammation [29]. However, surprisingly few studies have used iBCECs in determining the permeability of novel therapeutics in treating brain cancers [30]. Previously, microfluidic-based studies have shown that Temozolomide can induce apoptosis in GBM cells in the presence of a BBB while other eight clinically approved compounds showed an inability to cross an intact BBB [31]. It is reported that extracellular vesicles derived from GBM promote and increase BBB permeability [32] with interrupted expression of claudin-5 and occludin in BCECs [33–35]. We have observed similar results in our set up, including significant reduction in TEER. Although there is a significant reduction in TEER, the measured values in the co-cultures remained to be above 500 Ω*cm^2^. In future studies more predominant investigations such as inclusion of electron microscopy and determination of paracellular substance transport will be required to examine the TJ disruptions of the BTB in detail. GLUT-1 is the most common glucose transporter in humans and is used as a valuable prognostic biomarker in tumors [38]. Due to the increased need of glucose uptake in hypoxic tumors, glucose is transported from blood to tumor site via the endothelium. Therefore, we aimed to investigate changes in GLUT-1 expression in iBCECs. Previous studies combining rat BCECs and glioma cells showed enhanced GLUT-1 expression attributed to increased VEGF secretions [39]. In our study, we were able to identify altered GLUT-1 expression in iBCECs upon co-culture with glioma cells.

One of the most widely accepted arguments to explain BBB disruption is the relatively high amount of VEGF secreted by the tumor mass. Generally, VEGF is overexpressed in GBM and is responsible for crosstalk between the tumor and the BCECs to promote angiogenesis [35, 36]. Our studies confirmed this observation, both GBM cell line-derived spheroid cultures were characterized by high VEGF secretion profiles. This seems to be one trigger for induction of barrier breakdown in our BTB test system [37].

Our results indicate successful formation of a BTB in-vitro test system, which can be established in short term and in a simplified manner. However, the model can be easily modified and adapted for different applications by inclusion of other cell types of the NVU, such as astrocytes, to increase model complexity as well as applicability. Giving the possibility to study astrocyte activation, shrinkage of astrocytic endfeet and disruption of the basement membrane in addition to endothelial barrier breakdown.

## Conclusions

We present a predictive and simplified in-vitro BTB test system by combining hiPSC-derived BCECs and GBM spheroids in a cell culture insert-based system. The BTB model reflects the in-vivo pathophysiology of the BBB endothelium induced by GBM including barrier disruption, altered expression of TJ proteins and GLUT-1 as well as increased VEGF secretion. By efficacy testing of standard GBM chemotherapeutics, showing the relevance of applying physiological 3D cultures in in-vitro testing, we demonstrated the applicability of the model system in preclinical research.

## Data Availability

No datasets were generated or analysed during the current study.
